# Cognitive Processes and Resting-State Functional Neuroimaging Findings in High Schizotypal Individuals and Schizotypal Personality Disorder Patients: A Systematic Review

**DOI:** 10.3390/brainsci13040615

**Published:** 2023-04-04

**Authors:** Chrysoula Zouraraki, Penny Karamaouna, Stella G. Giakoumaki

**Affiliations:** 1 Laboratory of Neuropsychology, Department of Psychology, University of Crete, 74100 Rethymno, Greece; zouraraki@uoc.gr (C.Z.); psyp211@psy.soc.uoc.gr (P.K.); 2 University of Crete Research Center for the Humanities, The Social and Education Sciences (UCRC), University of Crete, Gallos University Campus, 74100 Rethymno, Greece

**Keywords:** schizotypy, schizotypal traits, schizotypal personality disorder, schizophrenia spectrum, functional neuroimaging, fNIRS, fMRI, resting-state fMRI, systematic review

## Abstract

Ample research findings indicate that there is altered brain functioning in the schizophrenia spectrum. Nevertheless, functional neuroimaging findings remain ambiguous for healthy individuals expressing high schizotypal traits and patients with schizotypal personality disorder (SPD). The purpose of this systematic review was to identify patterns of task-related and resting-state neural abnormalities across these conditions. MEDLINE-PubMed and PsycINFO were systematically searched and forty-eight studies were selected. Forty studies assessed healthy individuals with high schizotypal traits and eight studies examined SPD patients with functional neuroimaging techniques (fNIRS; fMRI; Resting-state fMRI). Functional alterations in striatal, frontal and temporal regions were found in healthy individuals with high schizotypal traits. Schizotypal personality disorder was associated with default mode network abnormalities but further research is required in order to better conceive its neural correlates. There was also evidence for functional compensatory mechanisms associated with both conditions. To conclude, the findings suggest that brain dysfunctions are evident in individuals who lie along the subclinical part of the spectrum, further supporting the continuum model for schizophrenia susceptibility. Additional research is required in order to delineate the counterbalancing processes implicated in the schizophrenia spectrum, as this approach will provide promising insights for both conversion and protection from conversion into schizophrenia.

## 1. Introduction

**Figure 1:**
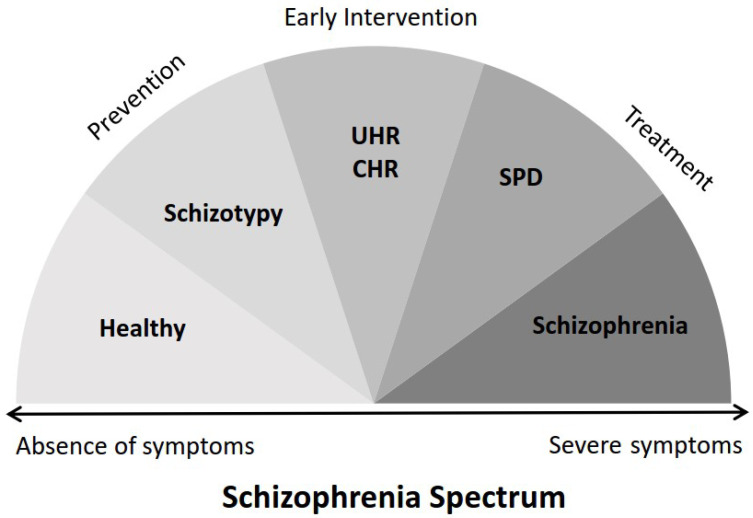
The Schizophrenia Spectrum. Abbreviations: UHR = Ultra High Risk, CHR = Clinical High Risk, SPD = Schizotypal Personality Disorder.

The term schizophrenia spectrum refers to a conceptual continuum [1] that differentiates schizophrenia-related phenotypes according to the number, severity and duration of symptoms (Figure 1). At the left end of the continuum, healthy individuals are positioned while at the right extreme end lies schizophrenia, which is a chronic neuropsychiatric debilitating disease, characterized by positive and negative symptoms, cognitive impairment, neuroanatomical and functional brain alterations [2]. Schizotypal personality disorder (SPD) precedes schizophrenia in the continuum and refers to an intermediate schizophrenia-spectrum phenotype [3] marked by reality distortion, negative affectivity, disorganization and impaired interpersonal functionality, but at a milder degree compared with schizophrenia [4]. Schizotypy is positioned one step prior to SPD [5] and is a multifaceted latent personality construct reflecting subclinical psychotic manifestations that are common in the general population [6]. Thus, schizotypy describes an endophenotype of schizophrenia [7] indicating proneness to related disease states [8]. Schizotypal traits are commonly assessed with self-report questionnaires/scales [9,10,11] and a similar factor structure between schizophrenia symptoms and schizotypal traits has been confirmed [12]. To this end, a common three-factor model categorizes schizotypal traits into positive, negative and disorganized [13,14,15] while a more analytical four-factor model further divides positive schizotypy into paranoid and cognitive-perceptual [16,17,18].

Several lines of research have pointed out the overlap between schizotypy, SPD and schizophrenia [19,20], indicating commonalities in genetic, neurobiological and psychosocial etiological factors [15,21,22,23,24]. These conditions are also characterized by analogous alterations in brain function [25,26,27,28] and qualitatively similar neurocognitive [29,30,31,32] and social cognition [3,33,34] impairments.

Despite recent advances in the field of schizophrenic pathophysiology, the need to improve the prediction of illness outcome using, among other variables, functional neuroimaging methods still exists [2]. With regard to this, previous reviews of functional neuroimaging studies have reported that schizophrenia patients present with task-related frontotemporal abnormalities [2], with alterations in the prefrontal cortex (PFC) and superior temporal gyrus being evident from the onset of the disease [35]. According to Attademo et al. [25], SPD patients show similar but milder dysfunctions of brain circuits (including striatal, frontal, temporal and limbic regions) compared with schizophrenia patients. Interestingly, individuals with high psychometric schizotypal traits also present with functional changes in frontal and temporal regions [28].

Parallel to the above, converging evidence from resting-state functional connectivity studies indicate that schizophrenia patients show abnormalities within and between regions of a cortico-cerebellar-striatal-thalamic loop [36]. Interestingly, striatal and default mode network (DMN) measures are predictors of antipsychotic response in individuals with schizophrenia spectrum disorders [37]. On the other hand, SPD patients show greater thalamo-frontal connectivity than patients with schizophrenia and this has been associated with milder symptom severity, indicating that this pattern may serve as a protective factor [38]. Based on a recent systematic review [28], individuals with increased schizotypal traits show both increased and reduced striato-cortical connectivity; however, clear patterns of functional connectivity changes associated with specific schizotypal dimensions were not identified.

The present systematic review focuses on studies assessing individuals with increased schizotypal traits and SPD patients with functional neuroimaging methods, in order to examine (a) the neuroanatomical characteristics of the SPD patients and healthy individuals with high schizotypal traits and (b) the cognitive correlates of neuroanatomical features of the SPD patients and healthy individuals with high schizotypal traits. This set of studies allow (a) the better conception of the neural correlates of the schizophrenia spectrum by avoiding the effects of confounding variables (e.g., medication, hospitalization, comorbidities) that affect schizophrenia patients and (b) the identification of compensatory-neuroprotective changes in brain functioning. Apart from the widely applied fMRI and rs-fMRI, the present study also included studies examining participants with Functional Near-Infrared Spectroscopy (fNIRS), the results of which were recently identified as potential clinical biomarkers for schizophrenia [39]. The findings are also presented separately for SPD and high schizotypal individuals, as this contributes to the formulation of a clearer view of the neural substrates implicated in these two conditions. Therefore, the aim of the present systematic review is to identify patterns of task-related (as indicated with fMRI and fNIRS) and resting-state (as indicated with rs-fMRI) neural abnormalities across SPD patients and healthy individuals with high schizotypal traits.

## 2. Materials and Methods

### 2.1. Review Question and Literature Search/Information Sources

Systematic literature searches of MEDLINE-PubMed and PsycINFO were completed between July and August 2022, following the guidelines of the Preferred Reporting Items for Systematic Reviews and MetaAnalyses-PRISMA [40]. The specific databases were chosen as per the recommendations of Löhönen et al. [41], who reported that these are the most inclusive databases for neuroimaging and schizotypal personality studies. The search string applied in both databases was: (‘fMRI’ OR ‘functional mri’ OR ‘functional neuroimaging’ OR ‘BOLD’ OR ‘fnirs’ OR ‘functional near-infrared spectroscopy’) and (‘schizotypal personality disorder’ OR ‘SPD’ OR ‘schizotypal’ OR ‘schizotypy’). Based on the ancestry approach, previous related reviews [25,28,42,43] were also examined for studies not identified in the literature searches.

### 2.2. Eligibility Criteria

**Figure 2:**
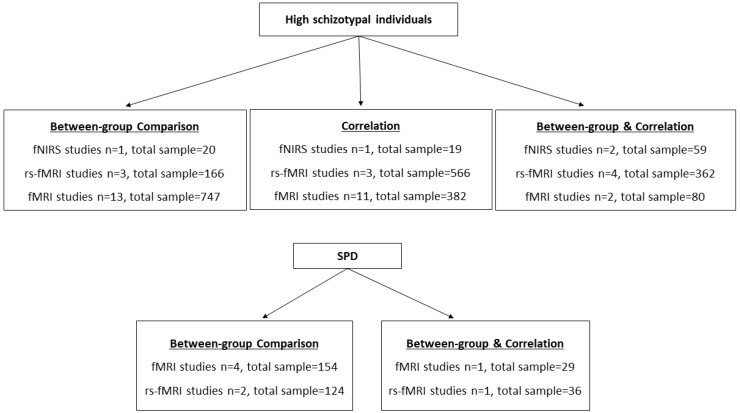
Total sample and number of between-group comparison and correlation studies included in the systematic review.

The selected studies reported either findings on the neuroanatomical characteristics or the cognitive correlates to neuroanatomical features of healthy individuals with high schizotypal traits and SPD patients and had to meet specific criteria. These included being published in peer-reviewed journals and written in English, reporting original empirical research, evaluating individuals using self-report questionnaires for schizotypal traits or clinical interviews for SPD, having a cross-sectional design with either between-group comparisons or correlational analyses (Figure 2), including a group with low schizotypal traits or a healthy control group as a comparison group (for studies with between-group comparisons), reporting functional neuroimaging data acquired with fMRI or rs-fMRI or fNIRS techniques and either administering a neuropsychological cognitive task or examining resting-state activity.

Two reviewers (CZ and PK) independently assessed the titles, abstracts and full texts of all the studies that met the inclusion criteria. The agreement rate was 84.38% and achieved consensus on which studies to include through discussion. Studies were excluded if (a) they assessed individuals with comorbidity with other psychiatric disorders, schizophrenia patients, relatives of patients, ultra-high-risk participants, or drug users, (b) used techniques other than those specified in the inclusion criteria (i.e., structural MRI, PET, EEG, SPECT), (c) did not evaluate schizotypy or SPD, (d) did not include a control group for the between-group studies, (e) were reviews or meta-analyses, dissertations, abstracts presented at meetings and letters to the editor.

### 2.3. Data Collection and Extraction

Two reviewers performed data extraction in duplicate, following the Population-Intervention-Control/Design-Outcome ‘PICO’ model [44]. The ‘Population’ were schizotypal individuals and SPD patients; the ‘Intervention’ included the assessment of participants with functional neuroimaging techniques; the ‘Comparison group’ was either a healthy control group or individuals with low scores on schizotypy measures; ‘Outcome’ was the functional neuroimaging findings and ‘Design’ referred to either categorical (between-group) comparisons or dimensional (correlational) analyses.

### 2.4. Quality Assessment of Studies

In order to assess the risk of bias in individual studies that were included in the systematic review, the adapted version of the Newcastle–Ottawa Scale (NOS) [45] for cross-sectional studies [46] was used. Two reviewers (CZ and PK) rated three different areas of biases, which are scored on a scale of 10 points: (1) selection of groups, which includes criteria such as recruitment strategy, response rate, representativeness of sample, validation of measurement tool; (2) comparability of the groups, which involves controlling for different confounders in analyses and (3) the outcome of the groups, which requires appropriate statistical analyses and outcome objectivity. For this systematic review, a minimum of 16 participants was considered a representative sample, as proposed by Friston [47]. The major confounding variable was the correction of the head motion for the fMRI studies and the respiration and cardiac artifacts for the fNIRS studies, whereas additional confounding factors were the age, gender, education, intelligence and depression scores of participants. Following the categorization suggested by Peng et al. [48], studies obtaining 0–4 points have low quality, 5–7 points indicate moderate quality and 8–10 points suggest high quality. The rate of agreement was 87.5 % and 100% consensus was achieved with discussion.

## 3. Results

### 3.1. Characteristics of the Included Studies

**Figure 3:**
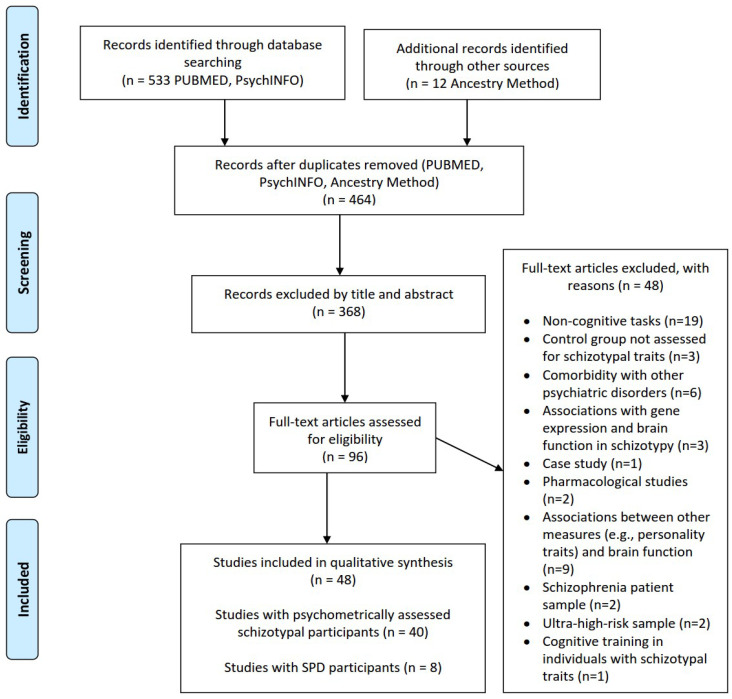
Flowchart of information on the selection of studies.

Initially, 533 records were found in the database search and an additional 12 records were discovered using the ancestry approach, resulting in a total of 545 records. After removing duplicates, 464 records were examined. Based on the eligibility criteria, a total of 48 studies were included in the qualitative synthesis, as shown in Figure 3. Among these studies, eight investigated SPD patients (*n* = 167, males/females 131/36, mean age 33.64) and healthy controls (*n* = 176, males/females 123/53, mean age 31.21). The remaining 40 studies focused on assessing schizotypal traits in college and/or healthy/community samples (with a total of 2400 participants). Among these studies, 26 had a between-group comparison design (with a total of 1463 participants) and provided data on high schizotypal individuals (*n* = 707, 290/391 males/females, mean age = 23.77) and low schizotypal individuals/controls (*n* = 756, 317/409 males/females, mean age = 24.88). One study with 56 participants did not provide information on the participants’ mean age or gender. Approximately half of the studies recruited college students (*n* = 21) and 27 studies included a community sample. A detailed graphical presentation of the study samples is also provided in Figure 2.

Table 1 and Table 2 provide information on the characteristics of the selected studies for individuals with high schizotypal traits and SPD patients, respectively. The tables include the following data items: (a) authors and year of publication; (b) sample size; (c) information on whether the study included high schizotypy/SPD and low schizotypy/control groups, along with their mean age and standard deviation; (d) gender ratio (males/females); (e) sampling recruitment strategy (i.e., community/college students/SPD patients); (f) study design (between-group comparisons or correlational analyses); (g) measures of schizotypal traits; (h) functional neuroimaging technique and the system used; (i) cognitive assessment; (j) findings for each study.

### 3.2. Results of Quality Assessment

The quality assessment results of the included studies are available in Supplementary Table S1. According to the modified Newcastle–Ottawa Scale for cross-sectional studies, the median quality score was 9 out of 10 points (range 7–10), indicating a high level of methodological quality overall. However, six out of forty-eight studies were considered moderate quality, primarily due to insufficient information regarding the sample’s representativeness and response rate. Despite this, none of the studies were excluded based on the quality assessment.

### 3.3. Functional Near-Infrared Spectroscopy (fNIRS) Studies

#### Cognitive Correlates of Neuroanatomical Features of High Schizotypal Individuals

Four studies examined the functional neuroanatomical substrate of schizotypy with fNIRS (total number of participants *n* = 98, high schizotypy group *n* = 40, low schizotypy/control group = 39, one study with correlational design *n* = 19) while participants completed executive functions and creativity tasks. Three studies assessed schizotypal traits with the SPQ and one study assessed schizotypy with the Oxford Schizotypal Personality Scale (STA) [96]. No fNIRS studies in SPD patients were identified.

Folley and Park [49] assessed creativity with a divergent thinking task and found increased right PFC activation for the high schizotypy group compared with controls. Regarding the activation patterns during a verbal fluency task (VFT), it was found that, in the letter version of the task, the high schizotypal group showed higher bilateral PFC activation and sustained PCF activation even during the post-task period, whereas the low schizotypy group showed a predominantly left PFC activation [51]. In both the category and letter versions of the VFT task, the high schizotypal group had higher right and lower left PFC activation compared with the low schizotypy group. This finding was also confirmed by a positive association between schizotypy scores and right prefrontal dominance in the letter condition of the VFT [50]. There was also a positive association between all three SPQ factor scores, odd speech and social anxiety scores with the average activation of the four right channels, whereas the unusual perceptual experiences subscale score was positively associated with average activation of the four right and left prefrontal (BA 10, BA 46 areas) channels [51]. Kobayashi et al. [52] did not find significant effects of schizotypal traits on PFC activation patterns during a fist-edge-palm (FEP) test.

### 3.4. Resting-State fMRI Findings

#### 3.4.1. Neuroanatomical Features of High Schizotypal Individuals

Ten studies reported resting-state functionality results (total number of participants *n* = 1095, high schizotypy group *n* = 240, low schizotypy/control group *n* = 289, number of participants in studies with correlational design *n* = 566). Participants were either university students or community samples. The majority of studies assessed schizotypal traits with the SPQ (*n* = 6), two studies with the Social Anhedonia Scale [9,97,98], one study with the Unusual Perceptual Experiences subscale of the Oxford–Liverpool Inventory of Feelings and Experiences (O-LIFE) questionnaire [10] and one study used the Psychotic-Like Experiences Battery (PLEs, including measures from O-LIFE [10], Chapman’s scales [9,99,100], Peter’s Delusion Inventory (PDI) [101] and Community Assessment of Psychotic Experiences [102]).

##### Total Schizotypy

Participants with high total SPQ scores had reduced functional connectivity (FC) between (a) the hippocampus and left dorso-caudal putamen, right caudate, left thalamus [60], (b) left insula and left putamen [53] and (c) sub-regions of the auditory, sensorimotor, visual, task control and default mode networks [62] compared with the control group. On the other hand, high SPQ scorers had increased FC between (a) the left declive of the cerebellum and right medial frontal gyrus [53], (b) the DMN and the salience and executive control networks [58] and (c) frontoparietal and auditory networks [62]. Studies with a correlational approach revealed that total SPQ scores were associated with (a) lower FC between the right dorsal caudate and posterior cingulate as well as left ventral rostral putamen and right superior frontal gyrus; (b) higher FC between the right ventral rostral putamen and superior frontal gyrus as well as left ventral rostral putamen and cingulate and (c) a higher asymmetry index of the right ventral rostral putamen [55]. One study did not report significant findings [61].

##### Negative Schizotypy

Individuals with high Social Anhedonia displayed lower FC between the posterior cingulate cortex and bilateral NAc [54] and between the hippocampal formation and parahippocampal cortex [59]. In addition, high FC was found between the frontal gyrus (medial and superior) with bilateral NAc and dorsorostral putamen, as well as between the insula and ventral caudate [54]. Increased FC was also reported in the interconnections of the retrosplenial cortex with insula and medial frontal gyrus and between the parahippocampal cortex and medial frontal gyrus [59]. High scores on the PLEs negative dimension were positively correlated with FC between the dorsocaudal putamen and right primary motor area [56].

##### Positive Schizotypy

High Unusual Perceptual Experience scorers had lower FC between (a) ventral striatal regions and ventromedial PFC, (b) ventrorostral putamen and frontal areas (medial orbital gyrus, left gyrus rectus, right ACC) and (c) dorsal striatal regions (dorsolateral putamen) and temporal-occipital areas (hippocampus, occipital gyrus, calcarine sulcus, cerebellar regions) [57]. Moreover, participants with high cognitive-perceptual SPQ scores showed higher FC between the hippocampus, thalamus and caudate compared to the control group and these findings were also confirmed using correlational analyses [60]. High scores on the positive dimension of PLEs were negatively associated with FC between (a) the dorso-rostral putamen and right DLPFC, (b) the dorsal caudate and left dorsal ACC and (c) the dorsocaudal putamen and right primary motor cortex [56]. Cognitive-perceptual SPQ scores were positively associated with FC between the right ventral rostral putamen and right middle frontal gyrus and inferior parietal lobe as well as the left ventral rostral putamen and right medial frontal gyrus [55]; they were also negatively correlated with FC between the left middle occipital gyrus and left inferior parietal lobule within the high schizotypal group [62].

##### Disorganized Schizotypy

Disorganized schizotypy, as assessed with the SPQ, was negatively associated with FC between (a) the right dorsal caudate and posterior cingulate, (b) the left dorsal caudal putamen and left cuneus and (c) the right dorsorostral putamen and middle temporal gyrus [55].

#### 3.4.2. Neuroanatomical Characteristics of SPD Patients

Three studies examined SPD individuals (total number of participants *n* = 160, SPD *n* = 82, controls *n* = 78) with rs-fMRI. According to Szeszko et al. [38], SPD individuals with low SPQ scores (according to a tercile split in SPQ Total score) had higher FC between the mediodorsal nucleus of the thalamus and rostral middle frontal cortex compared with individuals with high SPQ scores. Schizotypal personality disorder patients showed increased DMN FC between (a) the superior temporal gyrus with putamen and caudate areas (anterior component of DMN) and (b) bilateral posterior cingulate gyrus (posterior component of DMN) compared with controls [94]. On the other hand, they had decreased DMN FC in (a) the medial frontal gyrus and anterior lobe of the cerebellum (anterior component of DMN) and (b) the posterior cerebellar lobe, right traverse temporal gyrus and left middle temporal gyrus (posterior component of DMN) compared with the control group [94]. Finally, SPD patients displayed lower FC between the right precuneus with parahippocampus and right middle temporal gyrus, right parahippocampus and right superior temporal gyrus and higher FC between the right precuneus and right middle frontal gyrus compared with controls [95].

### 3.5. fMRI Studies

#### 3.5.1. Cognitive Correlates of Neuroanatomical Features of Schizotypal Individuals

Twenty-six studies assessed individuals with schizotypal traits while performing tasks of social cognition, executive functions, memory, learning and creativity (total number of participants *n* = 1221, high schizotypy group *n* = 426, low schizotypy/control group *n* = 443, total number of participants in studies with a correlational design *n* = 352).

##### Social Cognition

Ten studies assessed emotion processing in high schizotypal individuals: five studies administered facial emotion processing tasks [68,79,83,85,88], one study an auditory emotion processing task [87], one study a dynamic facial expression processing and social interaction task [71] and three studies used tasks of emotion processing while viewing affective stimuli/pictures [64,65,82].

In the facial emotion processing tasks, individuals with high social anhedonia were compared with low social anhedonia participants and were reported to have (a) reduced activation in the right superior frontal gyrus and right superior temporal gyrus [68], (b) increased activation in the bilateral thalamus and left red nucleus [83] and (c) reduced neural connectivity between the left ventral-lateral prefrontal cortex (VLPFC) and left inferior parietal cortex, precentral gyrus, bilateral inferior temporal sulcus and right superior temporal sulcus [79]. One study examined both social and physical anhedonia [85] and found that individuals with high negative schizotypy had lower activation in the medial prefrontal cortex (mPFC) and the amygdala in the neutral and the fearful conditions of a facial emotional discrimination task compared with the low negative schizotypy group. In addition, the high negative schizotypy group had reduced functional connectivity between the amygdala and mPFC/dorsal anterior cingulate cortex in the happy and fearful conditions of the task. Finally, a positive association between SPQ disorganized factor score and activation in the right posterior superior temporal sulcus during a neutral face processing task was reported by Yan et al. [88]. The processing of dynamic happy facial expressions under different social interaction cues (i.e., praise and blame cues) was assessed by Huang et al. [71]. They found that participants with a high total SPQ score showed decreased activation in the left cingulate cortex and right superior temporal gyrus in the blame cues and increased activation in the right anterior cingulate cortex in the happiness disappearing facial expressions condition of the task.

Olano et al. [87] examined the association between emotion processing using an auditory emotional task and the O-LIFE total score in a student sample. They reported that the total score positively correlated with activation in the right orbitofrontal cortex, right anterior cingulate cortex and left medial temporal gyrus; activation in the latter two regions also correlated with the unusual experiences subscale during a low intelligibility condition of the task (i.e., the degradation of the auditory signal).

Emotion processing while viewing affective stimuli was examined in three studies. According to Modinos et al. [82], participants scoring high on the unusual experiences subscale of O-LIFE showed increased activity in the caudate while viewing emotional pictures compared to low scorers. During positive stimuli processing, physical anhedonia was (a) negatively correlated with activation in the left inferior frontal gyrus [64] and left medial PFC, left inferior and right middle temporal gyri, left cuneus, right superior parietal gyrus and right anterior cingulate [65] and (b) positively correlated with activation in the ventral medial prefrontal cortex (VMPFC), right middle temporal gyrus, left superior temporal gyrus, right insula, right superior parietal lobule and right occipital lobe [64]. During the processing of negative stimuli, physical anhedonia was positively correlated with activation in the bilateral middle temporal gyri, right superior parietal lobule, left supramarginal gyrus and right cuneus [64].

Three studies examined another aspect of social cognition by administering Theory of Mind (ToM) tasks in student samples. Wang et al. [78] reported a positive correlation between social anhedonia and activation in the right cuneus, bilateral middle temporal gyrus, medial frontal gyrus and right temporo-parietal junction. Additionally, physical anhedonia correlated positively with activation in the left middle temporal gyrus. Individuals scoring high on the positive subscale of the Community Assessment of Psychic Experiences questionnaire (CAPE) [102,103] showed hyperactivation of the anterior PFC, lateral PFC bilaterally and right dorso-medial PFC during second order mentalizing conditions (i.e., conditions requiring the attribution of a cognitive or affective mental state) [66]. One study focused on self-perspective inhibition, which is a necessary ToM component for understanding the mental states of other people [72], and reported that individuals scoring high on the positive scale of CAPE had increased activation in the left inferior frontal gyrus compared to the low scorers.

One study examined irony comprehension in a sample from the general population [67]. They reported that the SPQ total score correlated positively with activation in the left inferior frontal gyrus and negatively with activation in the middle temporal gyrus bilaterally and the right superior occipital gyrus during the irony comprehension condition of the task. Specific associations between SPQ factor scores and brain pattern activations were also found: the cognitive perceptual factor score was negatively associated with activation in the middle temporal gyrus and positively with the right superior frontal gyrus; the interpersonal factor score had a positive association with activation in the right precentral gyrus, left thalamus and right inferior occipital gyrus.

Schmidt et al. [86] assessed brain activation during a task examining the tendency for social jumping to conclusions and reported a negative correlation between constricted affect and nucleus accumben (NAc) activation. Finally, Hooker et al. [74] examined social reward processing in healthy individuals with high social anhedonia and reported that they hypo-activated the ventral-lateral prefrontal cortex (VLPFC), posterior insula, superior frontal and temporal gyrus and mPFC compared with a low social anhedonia group during the positive social cues condition.

##### Memory and Learning

Corlett and Fletcher [69] examined neural responses in a Kamin blocking task (i.e., learning causal relationships between foods and allergic reactions) in healthy individuals assessed with Chapman’s scales [9,99,100] and PDI [104]. There were negative associations between (a) high magical ideation scores and the magnitude of striatal activation during the prediction of the error signal (i.e., individuals have not learned the blocked cue) and (b) the PDI distress scores with activation in the frontal cortex, striatum and midbrain during the prediction of error response to the violation of blocking expectation (i.e., individuals who did not learn the blocked cue had high distress scores). There was also one positive association between the PDI distress score and inappropriate DLPFC responses during blocking trials. Ettinger et al. [70] assessed individuals from the general population with the Eysenck Personality Questionnaire (EPQ) [105] and a procedural learning task. The EPQ Psychoticism score correlated positively with the activity in three clusters during procedural learning: (1) the right transverse temporal gyrus extending to the putamen, caudate, thalamus and insula; (2) the inferior frontal and precentral gyri and (3) the middle frontal gyrus extending to the precentral gyrus and anterior cingulate. The Schizotypal Personality Scale (STA) [106] score correlated positively with activity in the right middle temporal gyrus. Wang et al. [75] assessed the neural correlates of schizotypal individuals during a prospective memory task and found that participants with a high SPQ total score had decreased activations in the inferior and medial frontal lobes.

##### Response Inhibition and Decision Making

Mohanty et al. [63] found that in the negative minus neutral condition of an emotional Stroop task individuals with high perceptual aberrations/magical ideation had (a) greater activation in the right DLPFC, right inferior frontal gyrus, right parahippocampal gyrus, left putamen and left cerebellum and (b) lower activation in the left DLPFC, left superior and right inferior temporal gyri and right middle occipital gyrus compared with controls.

Four studies examined brain activation patterns during tasks that examine the anticipatory and consummatory components of hedonic capacity in individuals with high schizotypal traits [77,80,81,84]. It was found that individuals with high negative schizotypy hypoactivate the right postcentral gyrus, left amygdala, left culmen and left putamen during gain consummation compared with controls. Additionally, during the gain anticipation condition, individuals with high positive schizotypy hyperactivated the right VLPFC and those with high negative schizotypy hypoactivated the left middle temporal gyrus, left ventral striatum and bilateral cerebellar tonsil compared with controls. Social anhedonia also correlated with higher brain activation patterns in the right anterior insula during gain anticipation in the schizotypy sample [81]. Individuals with high CAPE scores showed reduced activation in the right caudate head [84] and in the ventral striatum [77] during the anticipation phase of the task compared to the low CAPE group. The study of Chan et al. [80] did not find significant results for the Incentive Monetary Delay Task but reported significant differences between a social anhedonia group and controls in the activation of the left thalamus, left pulvinar and right insula during affective incentives.

##### Creativity

Two studies examined the association of neural activation patterns with schizotypal traits while participants completed creativity tasks [73,76]. High SPQ scorers showed stronger activation in the left superior temporal gyrus and the right precuneus and lower activation in the anterior cingulate, left frontal and inferior parietal regions compared with low SPQ scorers during the alternative uses task [73]. Park et al. [76] found (a) negative correlations between O-LIFE unusual perceptual experiences and impulsive nonconformity and activation in the left frontal gyrus, left inferior parietal lobule and right inferior temporal gyrus and (b) a positive correlation between introvertive anhedonia and activation in the right middle occipital gyrus during the Torrance Tests of Creative Thinking.

#### 3.5.2. Cognitive Correlates of Neuroanatomical Features of SPD Patients

Five studies examined brain activation in SPD patients while performing tasks of social cognition and working memory (total number of participants *n* = 183, SPD patients *n* = 85, control individuals *n* = 98). Schizotypal personality disorder patients were recruited from clinical services and the community.

##### Social Cognition

Two studies examined emotion processing [90,91] and one study examined the approachability component of social judgement [93]. Thus, Dickey et al. [90] reported that SPD patients utilized mainly frontal areas and had less activation in the left superior temporal sulcus and the left insula during prosody identification. Schizotypal personality disorder patients also showed greater activation of the amygdala during affective picture processing compared with controls [91]. Finally, Stanfield et al. [93] did not find any significant differences in brain activation patterns between SPD patients and controls during a social judgement task.

##### Working Memory

Two studies assessed working memory employing a visual n-back task and a visuospatial task [89,92]. Overall, SPD patients showed reduced activation in several frontal and temporal regions compared with controls. While performing the 0-back condition of the n-back task, the SPD group showed decreased activation in the left procentral gyrus, whereas in the 2-back condition they also had reduced activation in the left posterior cingulate gyrus, left superior temporal gyrus and left middle frontal gyrus [92]. During the maintenance period of the visuospatial working memory task, SPD individuals also had decreased activation in the left vPFC, left superior frontal gyrus, left intraparietal cortex and the left posterior inferior frontal gyrus, while during the retention period of the task they showed decreased activation in the left superior temporal gyrus and left posterior inferior frontal gyrus compared with the control group [89].

## 4. Discussion

The aim of the present systematic review was to more completely formulate a frame of task-related and resting-state functional neural correlates of schizotypy and SPD. Forty-eight studies were examined and the comparison of brain activity patterns between individuals with high schizotypal traits or SPD patients and control individuals revealed some consistent findings: (a) individuals with high schizotypal traits and SPD patients present with a dysfunctional corticostriatal circuitry during resting-state and cognitive task performance, in accordance with the existing literature on the schizophrenia spectrum [107,108,109,110,111]; (b) there is preserved PFC activation or hyperactivation in schizotypy, which may either indicate the existence of frontal compensatory mechanisms that protect individuals from converting into schizophrenia or may reflect greater neural effort in these individuals in order to standardize their behavioral performance; (c) the altered activation of key brain regions (amygdala, frontal and temporal areas) implicated in social cognition is observed in both high schizotypal and SPD individuals and (d) there are DMN connectivity abnormalities in SPD patients during resting state.

### 4.1. fNIRS Studies

Studies employing fNIRS have focused mainly on brain activation patterns during verbal fluency or creativity-related tasks and support the existence of preserved PFC activation during intact behavioral performance in high schizotypal individuals [49,50,51]. Interestingly, the dominance of the right PFC in the high schizotypal groups indicates a qualitative similarity between schizotypy and schizophrenia, as both conditions are associated with a greater right than left asymmetry [112]. The one study that assessed brain activation while participants completed the widely used FEP task assessing sequential movement abilities did not report significant associations between high positive schizotypal traits and PFC hemodynamic responses [52]. Since the tuned and interactive functioning of cortical, subcortical and cerebellar areas is required in order to successfully execute the required movements [113], a plausible explanation for these findings is that other brain areas rather than the PFC mediate FEP task performance in positive schizotypal individuals [52].

### 4.2. rsFMRI Studies

Resting-state fMRI findings overall describe abnormal striatal FC in individuals with high schizotypal traits, in accordance with evidence in other populations falling in the schizophrenia spectrum, i.e., in patients with schizophrenia spectrum disorders [114], first episode schizophrenia patients [115] and individuals at risk for psychosis [107]. Apart from its key role in movement-mediating neural circuitries [116], the striatum also has extensive neuroanatomical connections with cortical and subcortical regions, thus modulating complex cognition and behavior [117,118] and being implicated in schizophrenia psychopathology [119] as well as the pathogenesis of its cognitive symptoms [120]. In accordance with these observations and aligning with the schizophrenia spectrum, disturbed striatal FC in high schizotypal individuals could be potentially associated with the severity of their schizotypal traits and/or their cognitive deficits; this requires further investigation though. Interestingly, and contrary to the reduced striatal FC and its potential effects/associations with schizotypy, Wang et al. [58,62] highlighted the possibility that the increased FC strength between frontoparietal and auditory networks may serve compensatory effects in high schizotypal individuals, whereas the hyperconnectivity observed between default mode with salience and executive control networks may be associated with schizophrenia-like symptoms.

As far as the specific schizotypal dimensions are concerned, rs-fMRI findings suggest that (a) there is altered cortico-striatal connectivity in individuals with high positive schizotypy [55,56,57]; the association of positive schizotypy with lower FC between areas of visual, posterior default mode and task control networks could be the basis of abnormal perception, suspiciousness and self-referential thought [62]; (b) high negative schizotypy/social anhedonia is associated with abnormal striatal FC, which could be an early change in the reward system of the brain [54], similar to the hypo-connectivity also found in psychotic patients [114] and (c) the reduced FC of the dorsal striatum with the posterior cingulate and middle temporal gyrus is associated with disorganized schizotypy [55] in accordance with the contribution of these brain regions in cognitive impairment and general pathology in schizophrenia [121,122].

Results of rs-FMRI studies assessing SPD patients indicate an altered default mode network activity compared with control individuals [94,95], in line with findings in clinical high-risk individuals [123], first episode and chronic schizophrenia patients [124,125]. One study reported increased FC from the mediodorsal nucleus of the thalamus to the rostral middle frontal cortex in SPD patients with low SPQ scores versus patients with high SPQ scores [38] and the authors pertinently pointed out that there might be frontal mechanisms that shield SPD patients from severe deficits.

### 4.3. fMRI Studies

#### 4.3.1. Social Cognition

Functional magnetic resonance imaging findings of studies assessing brain activation during emotion processing suggest that high schizotypy [71], in particular anhedonia, is consistently associated with reduced neural response in key regions of emotion perception, regulation and processing [65,68,74,79,85], such as the amygdala and other temporal areas as well as different parts of the PFC. These findings support the “disconnectivity hypothesis” that postulates that schizophrenia pathophysiology results from a disruption of the connectivity between different brain regions [126,127,128] and are in accordance with studies reporting functional alterations in these regions in schizophrenia spectrum individuals [43,129]. Another interesting pattern of findings was that some brain areas involved in social cognition processes have been found to be overactive in high schizotypal individuals and this may set the basis for the emergence of clinical symptoms and schizophrenia vulnerability. In detail, (a) Günther et al. [83] reported that increased thalamus and red nucleus activation during the processing of negative affective stimuli reflects a heightened sensitivity to negative social cues, which may play a role in the avoidance of social interactions; (b) Yan et al. [88] proposed that the increased responsiveness of the posterior superior temporal sulcus to emotionally neutral stimuli is an endophenotype of schizophrenia and might be associated with hypermentalization and vulnerability to delusions; (c) Olano et al. [87] demonstrated that the overactivation of the right anterior cingulate cortex and left medial temporal gyrus in the most degraded condition of emotionally negative and neutral auditory signals implies that hearing irrelevant stimuli potentially captures the attention of high schizotypal individuals, which qualitatively resembles findings in schizophrenia patients with auditory verbal hallucinations [121,130] and finally (d) Modinos et al. [82] suggested that high striatum activity during emotion processing in individuals with high unusual perceptual experiences is associated with glutamate levels in some of the regions involved in emotion processing. Findings on ToM and irony comprehension processing, which are also components of social cognition, suggest that there is a positive association between high schizotypy and activation in frontal regions [66,67,72,78]. These studies provide one common interpretation of the findings: abnormalities on the brain functional level but not on the behavioral level suggest that high schizotypal individuals require greater effort to reach normal behavioral performance and this is reflected by the increased neural activation during the more complex inferences of ToM tasks. Since the reduced recruitment of frontal and temporal regions of the mentalizing network has been found in schizophrenia patients [131], the inverse pattern of activation in subclinical individuals indicates the existence of a possible neuroprotective mechanism. Only two studies [90,91] have found significant results in the activation pattern of SPD patients during emotion processing and reported increased activation in a fronto-temporal network that includes the parahippocampus and amygdala. Due to the scarcity of studies in the literature and the limitations of the available studies (i.e., differences in the medication status of patients and tasks employed, small sample sizes) it is difficult to reach specific conclusions, even though the results in SPD patients are in part similar to the aforementioned findings in high schizotypal individuals.

#### 4.3.2. Memory and Learning

The study by Corlett and Fletcher [69] also supports the continuum model of schizophrenia by reporting that facets of positive schizotypy (i.e., magical ideation/unusual beliefs) are associated with aberrant striatal functioning during a learning and memory task. Interestingly, the authors also reported that when unusual beliefs are accompanied by higher distress and PFC dysfunction, they set the basis for clinical delusions. Further advancing the literature on the neural correlates of learning and memory, Ettinger et al. [70] found an association between EPQ psychoticism and increased activation in a fronto-striato-thalamic circuitry, which is implicated in the dopaminergic dysfunction and symptom onset across the schizophrenia continuum [132,133] during the procedural learning of motor sequences. Finally, Wang et al. [75] found hypoactivation of the PFC but intact behavioral performance during a prospective memory task. Hypofrontality while performing a prospective memory task in schizophrenia patients has also been reported by Chen et al. [134] and a recent meta-analysis also showed impaired behavioral performance in schizophrenia during prospective memory tasks [135].

Two studies assessed brain activation in SPD patients during different working memory tasks [89,92] and common findings such as reduced superior temporal gyrus activation in SPD patients and intact behavioral performance compared with controls emerged. Even though superior temporal gyrus structural and functional abnormalities [121] and significant working memory deficits [136] have been reported in schizophrenia patients, these two studies examining SPD patients propose that the differential recruitment of brain regions may help them compensate [137] and could play a role in their comparable working memory performance with controls.

#### 4.3.3. Response Inhibition and Decision Making

The study by Mohanty et al. [63] reported that when individuals with high positive schizotypy completed a selective attention/response inhibition emotional Stroop task they showed a hemispheric asymmetry in DLPFC activation: they hyper-activated the right DLPFC and at the same time they hypo-activated the homologous brain region in the left hemisphere during the processing of negative stimuli. As the authors proposed, this finding potentially indicates biased attention to negative emotional stimuli accompanied by difficulties in engaging executive processes optimally. High positive schizotypy was also associated with increased right inferior frontal gyrus activity, an area associated with the ability to inhibit the processing of irrelevant stimuli [138], further highlighting the existence of compensatory mechanisms in schizotypy (i.e., in order to reach an adequate behavioral performance level, individuals recruit this area at a higher degree than controls). Finally, dysfunctional brain activation patterns in both subcortical and cortical brain areas have been associated with schizotypy. Thus, decreased striatal activation was consistently found in individuals with high CAPE scores [77,84] and those with high negative schizotypy [81] during reward anticipation. During reward consummation, decreased amygdala activation was found in negative schizotypal individuals [81] in line with findings in schizophrenia patients [139]. On the other hand, the hyperactivation of VLPFC in high positive schizotypy individuals during reward anticipation [81] again implicates compensatory mechanisms in subclinical individuals.

#### 4.3.4. Creativity

Both studies examining brain activation patterns during creativity tasks [73,76] proposed associations between the neural substrate underlying creativity and schizotypy. In this context, individuals with high schizotypal traits showed stronger activation of the right precuneus [73]—a key brain area for divergent thinking [140] and part of the DMN [141]—during creative cognition. Park et al. [76] further described this association by reporting negative correlations between schizotypal traits associated with either unusual perceptual experiences or impulsive/disinhibited behavior and the activation of several frontal, temporal and parietal cortical areas, thus “*…highlighting the possibility that these dimensions work in conjunction for maximum creative output*” (p. 104).

## 5. Conclusions

Overall, the findings of the reviewed studies suggest that there are functional alterations in individuals with high schizotypal traits or SPD in striatal, frontal and temporal brain areas in line with findings in the schizophrenia spectrum. The findings also support the dimensional model of schizophrenia, suggesting that functional abnormalities are evident even in subclinical individuals and SPD patients. A number of studies provided evidence on the existence of functional compensatory mechanisms associated with frontal areas or the recruitment of different brain areas during task performance in schizotypy and SPD, which may help to regulate cognition. However, it is important to note that the study of functional neuroimaging in schizotypy and SPD is still in its early stages. In terms of future studies, there is a requirement for more research on SPD, as the number of the available functional neuroimaging studies is small and there are several methodological limitations such as the medication status of patients, small sample sizes, heterogeneity in recruitment strategy (i.e., participants from university settings, clinical services, community). In addition, the real existence and practical use of the potential neurocompensatory mechanisms should be confirmed in future studies as they will provide insights into the conversion and progression of schizophrenia.

A limitation of the study, though, is that the protocol was not registered in PROSPERO before the literature search and that the methodological heterogeneity of the existing literature did not allow us to conduct a meta-analysis. Certain limitations of the selected studies should also be highlighted. First, the sample sizes of the existing studies are rather small: 29 out of 48 studies did not fulfill the criterion of a satisfactory sample size (i.e., a minimum of 16 participants) based on the qualitative assessment, possibly limiting the detection of between-group differences and reducing the statistical power of findings. Indeed, quite recently Szucs and Ioannidis [142] emphasized this point with regard to neuroimaging studies and indicated the requirement of power calculations. Second, there is great variability in the assessment instruments of schizotypy and significant heterogeneity in the cut-off values that are used for the selection of high and low schizotypal groups. The net result is a significant difficulty in the comparison of findings between studies and the delineation of associations between different schizotypal traits and brain function. Third, several studies (19 out of 48) assessed university/college samples, thus limiting the generalizability of findings due to the restricted age range and educational attainment of participants. Fourth, even though the majority of the studies used a 3 Tesla MRI scanner, there were seven studies with 1.5 Tesla, setting some limitations to the quality of data acquisition. Finally, a few studies reported uncorrected thresholds of *p* values, mainly due to their exploratory approach. This is a significant issue regarding the validity of results as, in order to control for false positive results, it is necessary to use statistical correction methods such as the Bonferroni, the AlphaSim or the false discovery rate (FDR) corrections.

## Figures and Tables

**Table 1:** Characteristics of the selected studies for individuals with high schizotypal traits.

fNIRS Studies								
Study (Year)	Participant N, Mean Age (SD), Gender (M:F)	Sample	Design	Schizotypy Assessment	Neuroimaging Modality, Task	System, Spectrometer	Findings between Group Comparisons	Findings Correlations/ Regressions
Folley and Park (2005) [49]	10 high SCT, mean age = 23.3 (1.6), 5:5 10 CG, mean age = 36.4 (3.1), 6:4	Community sample	Between-group comparisons	SPQ three-factor model SCT Mean SPQ Total Score = 41.5 (1.1) CG Mean SPQ Total Score = 19.3 (3.5)	fNIRS, “alternative uses” divergent thinking task (DTT)	Hitachi ETG-100, 24-channel spectrometer	SCT ↑ right PFC activation during DTT vs. CG (FDR corrected *p* < 0.002v)	
Hori, Ozeki et al., 2008 [50]	16 high SCT, mean age = 41.1 (11.8), 2:14 16 low SCT, mean age = 40.2 (10.1), 8:8	Healthy individuals	Between-group comparisons and correlation analyses	SPQ three-factor model Median split High SCT Total SPQ Score = 19.6 (7.3) Low SCT SPQ Total Score = 6.3 (2.6)	fNIRS, Verbal fluency Letter and Category task (VFT)	FOIRE-3000, 31-channel spectrometer	High SCT ↑ right and ↓ left PFC activation in VFT (*p* < 0.05)	Positive association SPQ total score-right PFC dominance in the letter and category VFT (all *p* values < 0.05)
Hori, Nagamine et al., 2008 [51]	14 high SCT, mean age = 33.8 (13.6), 0:14 13 low SCT, mean age = 43.9 (10.2), 0:13	Healthy individuals	Between-group comparisons and correlation analyses	SPQ three-factor model Median Split High SCT SPQ Total Score = 16.7 (7) Low SCT SPQ Total Score = 4.5 (2.8)	fNIRS, Verbal fluency task (VFT)-letter version	Hitachi ETG-100, 24-channel spectrometer	High SCT ↑ bilateral PFC activation Low SCT ↑ left PFC activation High SCT showed sustained PFC activation in the post-task period vs. low SCT (all *p* values < 0.05)	Positive correlation SPQ subscale unusual perceptual experiences-activation of the four right and four left PFC channels (both *p* values < 0.05) Positive correlations SPQ total score (*p* < 0.01), cognitive-perceptual (*p* < 0.01), disorganized (*p* < 0.05) and interpersonal (*p* < 0.05) factors and odd speech, social anxiety subscales (all *p* values < 0.05) with the average activation of the four right PFC channels
Kobayashi et al., 2021 [52]	19 healthy participants, mean age = 23.00 (0.30), 9:10	Healthy individuals	Correlation analyses	Oxford Schizotypal Personality Scale (STA) Mean STA score = 9.15 (1.43)	fNIRS, Fist-Edge-Palm (FEP) task and palm tapping task as a control task	Shimadzu Co., Ltd., OMM3000, five-channel spectrometer		non-significant findings (all *p* values > 0.17).
Resting-state fMRI studies								
Study (Year)	Participant N, mean age (SD), gender (M:F)	Sample	Design	Schizotypy assessment	Scanner, Strength	Findings between group comparisons	Findings correlations/regressions	
Wang, Yan et al., 2015 [53]	35 High SCT, mean age = 19.7 (1.1), 19:16 34 Low SCT, mean age = 20.1 (0.9), 15:19	College students	Between-group comparisons and correlation analyses	SPQ three-factor model High SCT SPQ Total Score> 10th percentile Low SCT SPQ Total Score bottom 50%	Siemens Trio, 3T	High SCT (a) ↓ FC left insula-left putamen, (b) ↑ FC left declive of cerebellum-right medial frontal gyrus, vs. Low SCT (AlphaSim correction both *p* values < 0.05)	No-significant findings (all *p* values >0.05).	
Wang et al., 2016 [54]	21 High SocAn, mean age = 19.3 (1.0), 10:11 30 Low SocAn, mean age = 19.3 (0.9), 15:15	Participants recruited from university	Between-group comparisons	Social Anhedonia Scale Scores 0.5 SD above or below the gender mean High SocAn Mean Total score = 15.19 (3.28) Low SocAn Mean Total Score = 3.07 (1.59)	Siemens Verio 3 T	High SocAn group ↓ FC posterior cingulate cortex-bilateral nucleus accumbens vs. Low SocAn group (*p* = 0.001) High SocAn group ↑ FC medial frontal gyrus-bilateral nucleus accumbens, insula-ventral caudate, superior frontal gyrus-dorsorostral putamen (all *p* values < 0.001)		
Wang, Ettinger et al., 2018 [55]	111 participants, mean age = 26.91 (7.9), 55:56	Healthy individuals	Correlation analyses	SPQ three-factor model Mean SPQ Total score = 7.81(6.72)	Siemens MAGNETOM Verio, 3T		Negative correlations SPQ total score—FC between (a) right dorsal caudate-bilateral posterior cingulate, (b) left ventral rostral putamen (VRP)-right superior frontal gyrus (all p_FWE_ values < 0.05) Positive correlations SPQ total score-FC between (a) right VRP-superior frontal gyrus, (b) left VRP-cingulate (all p_FWE_ values < 0.05) Positive correlations Cognitive-perceptual SPQ factor-FC between (a) right VRP-right middle frontal gyrus and inferior parietal lobe, (b) left VRP-right medial frontal gyrus (all p_FWE_ values < 0.05) Negative correlations Disorganized SPQ factor –FC between (a) right dorsal caudate-posterior cingulate, (b) left dorsal caudal putamen-left cuneus, (c) right dorsal rostral putamen-middle temporal gyrus (all p_FWE_ values < 0.05) Positive correlation between SPQ total score and asymmetry index of the right VRP (*p* < 0.001).	
Sabaroedin et al., 2019 [56]	353 participants, median age = 22, 155:198	Community sample	Correlation analyses	Online battery of Psychosis-Like Experiences (PLE) measures: 1. Short-form Oxford–Liverpool Inventory of Feelings and Experiences 2. Peters Delusion Inventory 3. Community Assessment of Psychotic Experience 4. Chapman Scales magical ideation, perceptual aberration, social and physical anhedonia	3T		Positive PLE Dimension Higher scores on the positive PLE dimension were associated with (a) ↓ FC dorsorostral putamen-right DLPFC (*p* = 0.002, threshold-free cluster enhancement-TFCE corrected) (b) ↓ coupling dorsal caudate-left dorsal anterior cingulate cortex (ACC) (*p* = 0.011, TFCE corrected) (c) ↓ coupling dorsocaudal putamen (DCP)-right primary motor cortex (*p* = 0.01, TFCE corrected) Negative PLEs Dimension Negative PLEs correlation with ↑ FC DCP seeds-right primary motor area (*p* < 0.001 TFCE corrected)	
Waltmann et al., 2019 [57]	19 High positive SCT, mean age = 26.37 (7.09), 10:9 20 Low positive SCT, mean age = 26.35 (5.47), 10:10	Healthy individuals	Between-group comparisons and correlation analyses	O-LIFE Short Version-Unusual Experiences factor High SCT mean Unusual Experiences Score = 11.42 (4.31) Low SCT mean Unusual Experiences Score = 0.75 (0.97)	General Electric Discovery MR750, 3T	High positive schizotypy group vs. low positive schizotypy group (a) ↓ FC ventral striatum- bilateral gyrus rectus and right medial orbital gyrus (cluster wise *p*_FWE_ = 0.037), (b) ↓ FC ventrorostral putamen-right medial orbital gyrus, left gyrus rectus and right ACC (cluster wise *p*_FWE_ < 0.001), vs. low positive schizotypy group (c) ↓ FC dorsolateral putamen-right hippocampus (cluster wise *p*_FWE_ < 0.001), left middle occipital gyrus (cluster wise *p*_FWE_ = 0.005), calcarine sulcus (cluster wise *p*_FWE_ < 0.001) (d) ↓ FC dorsocaudal putamen-right middle occipital gyrus/calcarine sulcus (cluster wise p_FWE_ < 0.001), left hippocampus (cluster wise *p*_FWE_ < 0.001), cerebellar areas (cluster wise p_FWE_ = 0.038)	Non-significant findings (all *p* values > 0.061)	
Wang et al., 2020 [58]	30 high SCT, mean age = 21.30 (1.44), 13:17 30 low SCT, mean age = 22.40 (2.06), 12:18	College students	Between-group comparisons	SPQ High SCT Mean SPQ Total Score = 47.57 (4.07) top 10th percentile Low SCT Mean SPQ Total Score = 12.53 (7.49)	GE, 3T	High SCT ↑ FC between (a) Default mode network (DMN)- Salience network, (b) DMN-Executive control network, vs. Low SCT (all *p* values < 0.001 alpha-sim correction)		
Zhang et al., 2020 [59]	40 SocAn (26 with available neuroimaging data) Mean age of SocAn individuals = 20.70 (4.52), 15:25 46 CG (29 with available neuroimaging data), mean age οf CG = 21.87 (2.61), 11:35	Participants from a large sample pool	Between-group comparisons	Chapman Social Anhedonia Scale High SocAn Total score > 1.5 SD above the mean Low SocAn Total Score < 10 (below sample mean)	General Electric, Tesla not reported Task-affective forecasting	SocAn ↑ FC vs. CG (a) retrosplenial cortex-bilateral insula, snf medial frontal gyrus, (b) parahippocampal cortex-medial frontal gyrus SocAn ↓ FC vs. CG Hippocampal formation-parahippocampal cortex (All *p* values < 0.05 FDR corrected)		
Kozhuharova et al., 2021 [60]	22 high SCT, mean age = 19.45 (1.05), 8:14 23 low SCT, mean age = 20.13 (2.10), 6:17	Student population	Between-group comparisons and correlation analyses	SPQ three-factor model Bottom and Top 10% deciles of SPQ High SCT SPQ Total Score > 41 Low SCT SPQ Total Score < 12	Siemens Magnetom TIM Trio, 3T	High SCT ↓ FC vs. low SCT hippocampus-left dorsocaudal putamen (p_FWE_ = 0.02), right caudate-left thalamus (p_FWE_ = 0.04)	Positive effect between the positive SPQ factor and FC hippocampus—caudate and hippocampus—thalamus (both p_FWE_ values = 0.02).	
Zhou et al.,2021 [61]	115 participants, mean age = 21.37 (2.53) 40% males (full set of neuroimaging data only for 102 participants)	Healthy individuals	Correlation analyses	SPQ three factor SPQ Mean Total Score = 34.46 (17.27)	GE 3T		non-significant findings (all *p* values > 0.21)	
Wang et al., 2022 [62]	87 high SCT, mean age = 21.17 (2.22), 38:49 122 CG, mean age = 21.62 (2.15), 38:84	College students	Between-group comparisons and correlation analyses	SPQ three-factor model High SCT SPQ Total Score > 41 (top 10th percentile) CG SPQ Total Score < 41	GE, 3T	High SCT ↓ FC in areas of interest involved in sensorimotor network, auditory network, visual network, DMN network, task control network vs. CG (all *p* values < 0.05 FDR corrected) High SCT ↑ FC between (a) left superior frontal gyrus (frontoparietal task network)-right rolandic operculum area (auditor network), (b) right superior frontal gyrus (DMN)-right medial superior frontal gyrus (DMN) (all *p* values < 0.05 FDR corrected)	Negative correlation Cognitive-perceptual SPQ factor-FC strength left middle occipital gyrus-left inferior parietal lobule in high SCT (*p* = 0.003 FDR corrected)	
fMRI studies								
Study (Year)	Participant N, mean age (SD), gender (M:F)	Sample	Design	Schizotypy assessment	Scanner, Strength, Task	Findings between group comparisons	Findings correlations/ regressions	
Mohanty et al., 2005 [63]	17 High POS SCT, mean age = 19.1 (1.9), 12:5 17 CG, mean age = 20.5 (3.9), 7:10	Undergraduate students	Between-group comparisons	PerAb and MagId Scales High POS SCT score 1.5 SD > mean on PerAb or MagId CG score 0.5 SD < mean on both PerAb and MagId	GE Signa, 1.5T, Emotional Stroop task	Negative vs. Neutral Condition High POS SCT ↑ right DLPFC, right inferior frontal gyrus, right hippocampus, parahippocampal gyrus, left putamen, left cerebellum vs. CG (all *p* values < 0.05 corrected per-voxel error rate) High POS SCT ↓ left DLPFC, left superior temporal gyrus, right inferior temporal gyrus, right middle occipital gyrus vs. CG (all *p* values < 0.05 corrected per-voxel error rate)		
Harvey et al., 2007 [64]	29 participants, mean age = 28.9 (7.9), 14:15 (available neuroimaging data only for 17 participants)	Community sample	Correlation analysis	Revised Physical Anhedonia Scale (PAS) Mean Total score = 12.2 (7.7)	1.5 T Siemens Sonata scanner Emotion processing		Condition Positive vs. neutral information processing positive correlation PAS Total Score-Ventral Medial PFC, right middle temporal gyrus, left superior temporal gyrus, right insula, right superior parietal lobule, right occipital lobe (all *p* values < 0.001) negative correlation PAS Total Score- left inferior frontal gyrus (*p* < 0.001) Negative vs. neutral information processing positive correlation PAS Total Score-bilateral middle temporal gyri, right superior parietal lobule, left supramarginal gyrus, right cuneus (all *p* values < 0.001)	
Harvey et al., 2010 [65]	26 participants, mean age = 30.7 (9.8), 13:13	Community sample	Correlation analysis	Revised Physical Anhedonia Scale Mean PAS score = 13.0 (8.5)	1.5 T Siemens Sonata Emotion Processing (identification)		Positive vs. neutral information processing Negative correlation PAS Total Score-left medial PFC, left inferior and right middle temporal gyri, left cuneus, right superior parietal gyrus (all *p* values < 0.001) negative correlation PAS Total Score-right anterior cingulate (*p* = 0.03)	
Modinos, Renken et al., 2010 [66]	18 high SCT, mean age = 19.8 (1.9), 10:8 18 Low SCT, mean age = 21.00 (2.8), 10:8	Undergraduate students	Between-group comparisons	Positive subscale of the Community Assessment of Psychic Experiences questionnaire (CAPE) High SCT > 75th percentile Mean CAPE positive score = 1.74 (0.13) Low SCT < 25th percentile Mean CAPE positive score = 1.12 (0.04)	Philips Intera, 3T, Theory of Mind task	Second Order Mentalizing Condition High SCT ↑ anterior PFC, lateral PFC bilaterally, dorsomedial PFC vs. Low SCT (all *p* values < 0.05, cluster level corrected for multiple comparisons)		
Rapp et al., 2010 [67]	15 participants, mean age = 28.1 (8.0), 0:15	Community sample	Correlation analyses	SPQ Mean SPQ Total Score = 14.5 (13.2)	Siemens TRIO, 3T, Irony comprehension task		Irony comprehension condition Positive correlations SPQ total score-left inferior frontal gyrus (*p* < 0.001) SPQ INT-right precentral gyrus, left thalamus, right inferior occipital gyrus (all *p* values < 0.001) SPQ CP-right superior frontal gyrus (*p* < 0.001) Negative correlations SPQ total score-middle temporal gyrus bilaterally, right superior occipital gyrus (all *p* values < 0.001). SPQ CP-middle temporal gyrus bilaterally, right middle occipital gyrus (all *p* values < 0.001) Literal comprehension condition: Positive correlations SPQ total score-right medial frontal gyrus (*p* < 0.001) SPQ INT-right superior frontal gyrus, right thalamus, right inferior occipital gyrus, right middle temporal gyrus, left inferior frontal gyrus, left caudate nucleus (all *p* values < 0.001) SPQ CP-left anterior cingulate (*p* < 0.001). Negative correlations SPQ total score-right superior/inferior parietal lobule (*p* < 0.001) SPQ CP-right superior parietal lobule, the middle temporal gyrus (all *p* values < 0.001) SPQ INT-language lateralization in middle temporal lobe (*p* < 0.05)	
Germine et al., 2011 [68]	15 High SocAn, mean age = 31.5 (10.7) 7:8 15 Low SocAn mean age = 32.5 (12.5) 7:8	Community sample and university students	Between-group comparisons	Revised Chapman Social Anhedonia Scale High SocAn Total Score > 16 for females, >19 for males (top 10%) Mean SocAn Total Score = 26.3 (6.6) Low SocAn Total Score < 7 for females and < 9 for males Mean SocAn Total Score = 3.7 (2.9)	3.0 T Siemens Trio Face emotion processing (emotion and identity discrimination)	Condition Emotion discrimination vs. Identity discrimination Low SocAn vs. High SocAn ↑ right superior frontal gyrus (*p*_FWE_ < 0.05) and right superior temporal gyrus (*p*_FWE_ < 0.05) Condition Emotion discrimination vs. object discrimination Low SocAn vs. High SocAn ↑ left superior frontal gyrus (*p*_FWE_ < 0.05) Condition Emotion discrimination vs. pattern discrimination Low SocAn vs. High SocAn ↑ right superior temporal gyrus (*p*_FWE_ < 0.05)		
Corlett and Fletcher 2012 [69]	18 participants, 10:8 (mean age or age range not reported)	Community sample	Correlation analyses	Chapman scales (PAS, SocAn, PerAb, MagId, PDI) Mean PDI Score = 5 (3.1) Mean MagId Score = 4.6 (3.5) Mean PerAb Score = 3.8 (4.6) Mean PAS Score = 8.8 (4.2) Mean SocAn Score = 5.6 (4.0).	Siemens Trio, 3T, Blocking of causal learning task		Negative correlation MagId score-striatal prediction error (PE) magnitude to the blocked cue (*p* < 0.05 FDR corrected) Negative association PDI distress score -PE response to violation of blocking-induced expectation in the frontal cortex, striatum and midbrain (all *p* < 0.05 FDR corrected) Positive association PDI distress score-inappropriate DLPFC responses during blocking trials (*p* < 0.05 FDR corrected)	
Ettinger et al., 2013 [70]	26 participants, mean age = 33.62 (13.21), 13:13	Community sample	Psychoticism scale of EPQ-R, STA EPQ Mean Psychoticism Score = 6.35 (3.63) Mean STA Score = 6.23 (3.64)	Correlation analyses	General Electric Signa, 1.5T, procedural learning task (PL, i.e., difference between the mean RTs to random and pattern trials)		Procedural Learning vs. Control Condition Positive correlations EPQ-R Psychoticism right transverse temporal gyrus extending to the putamen, caudate, thalamus and insula (cluster p_FWE_ = 0.001) EPQ-R Psychoticism- inferior frontal and precentral gyri (cluster p_FWE_ = 0.007) EPQ-R Psychoticism- middle frontal gyrus extending to the precentral gyrus and anterior cingulate (cluster p_FWE_ = 0.001) STA scores-right middle temporal gyrus (p_FWE_ = 0.005)	
Huang et al., 2013 [71]	14 High SCT, mean age = 22.3 (2.1) 7:7 14 Low SCT, mean age = 20.7 (0.46), 8:6	Community sample	Between-group comparisons	SPQ High SCT SPQ Mean Total Score = 45.79 (1.84) (top 10th percentile) Low SCT SPQ Mean Total Score = 11.07 (1.25) (lowest 50th percentile)	Siemens Trio A Tim 3T Dynamic happy facial expression processing and social interaction task	Happiness Disappearing Condition High SCT ↑ right anterior cingulate cortex vs. Low SCT (*p* < 0.05, AlphaSim corrected) Blame Social Interaction Cues Condition High SCT ↓ left cingulate cortex (*p* = 0.01 AlphaSim corrected) and right superior temporal gyrus vs. Low SCT (*p* < 0.01 AlphaSim corrected)		
van der Meer et al., 2013 [72]	18 High SCT, mean age = 19.7 (1.9), 10:8 19 Low SCT, mean age = 21.6 (2.6), 10:9	Undergraduate students	Between-group comparisons	Community Assessment of Psychic Experiences (CAPE) positive subscale High SCT score one SD above mean CAPE Positive Mean Score = 1.80 (0.15) Low SCT score below the sample mean CAPE Positive Mean Score = 1.12 (0.04)	Philips Intera, 3T ToM Stop Signal Task	Self-perspective inhibition condition High SCT ↑ left inferior frontal gyrus vs. Low SCT (*p* < 0.05 FDR corrected)		
Fink et al., 2014 [73]	21 High SCT, mean age = 23.29 (4.17), 9:12 20 Low SCT, mean age = 22.85 (3.42), 8:12	University students	Between-group comparisons	SPQ three-factor model High SCT SPQ scores range = 132–179 Low SCT SPQ scores range = 3–77	Siemens Tim Trio, 3T, Alternative-uses task for creative cognition (AU)	Alternative vs. common uses Low SCT ↑ left superior/middle frontal (*p*_FWE_ = 0.006, ηp2 = 0.19), left inferior frontal (p_FWE_ = 0.002, ηp^2^ = 0.25), left inferior parietal regions (*p*_FWE_ = 0.0004, ηp^2^ = 0.30), anterior cingulate (*p*_FWE_ = 0.001, ηp^2^ = 0.28) vs. High SCT. High SCT ↑ left superior temporal gyrus (*p*_FWE_ = 0.0001, ηp^2^ = 0.35), right precuneus (p_FWE_ = 0.04, ηp^2^ = 0.11) vs. Low SCT.		
Hooker et al., 2014 [74]	15 High SocAn, mean age = 32.00 (12.75), 7:8 15 Low SocAn, mean age = 30.27 (10.47), 5:10	Healthy individuals	Between-group differences	Revised Social Anhedonia Scale High SocAn score 1.96 SDs above population mean Mean SocAn Total score = 24.6 (5.63) Low SocAn score equal or less than 1 SD above the population mean Mean SocAn Total score = 2.67 (2.53)	Siemens Tim Trio, 3T, social reward task		Positive vs. Neutral Expressions High SocAn ↓ventral lateral prefrontal cortex (VLPFC), posterior insula, superior temporal gyrus, superior frontal gyrus vs. Low SocAn (all *p* values < 0.001) Positive vs. Negative Expressions High SocAn ↓rostral anterior cingulate cortex, middle cingulate cortex, posterior insula vs. Low SocAn (all *p* values < 0.001)	
Wang et al., 2014 [75]	19 SCT, mean age = 19.37 (1.07), 10:9 22 CG, mean age = 19.68 (0.72), 12:10	University students	Between-group comparisons	SPQ three-factor model SCT scores top 10% Mean SPQ Total Score = 39.53 (3.63) CG lowest 50% mean Mean SPQ Total Score = 16.64 (4.86)	Siemens Trio A Tim, 3T, Event-based prospective memory task	Prospective memory vs. baseline Condition SCT ↓ inferior frontal gyrus, medial frontal gyrus vs. CG (both *p* values < 0.001 AlphaSim corrected)		
Park et al., 2015 [76]	48 participants, mean age = 23.42 (4.50), 17:31	Participants recruited through university and online research recruitment websites	Correlation analyses	O-LIFE	Siemens Magnetom Skyra, 3T, figure and verbal creativity–drawing task		Create condition Negative Correlations UnExp-left superior medial frontal gyrus (*p* = 0.017), left middle frontal gyrus (0.013), left inferior parietal lobule *p* = 0.027), right inferior temporal gyrus (*p* < 0.001) O-LIFE ImpNon-left middle frontal gyrus (*p* = 0.028), the right inferior temporal gyrus (*p* < 0.001) Positive association O-LIFE IntAn-signal difference in Create and Trace conditions right middle occipital gyrus (*p* = 0.041).	
Simon et al., 2015 [77]	11 High CAPE, mean age = 28 (9), 3:8 14 Average CAPE, mean age = 26.4 (5.3), 6:8 12 Low CAPE, mean age = 25.5 (8.5), 4:8	Healthy individuals recruited using the internet	Between-group comparisons and correlation analyses	CAPE Low CAPE 1 SD < mean High CAPE 1 SD > mean Average CAPE a deviation of less than 1 SD from mean	1.5-Tesla Siemens Magnetom Avanto Cued Reinforcement Reaction Time task (anticipation of monetary gains and losses)	Anticipation of €2 compared with €0 condition High CAPE ↓ ventral striatum vs. Low CAPE (p_FWE_ = 0.04)	Anticipation of a Reward ventral striatal connectivity- medial PFC, right dorsal striatum, bilateral insula, left DLPFC (all *p* values < 0.05)	
Wang et al., 2015 [78]	56 participants, mean age = 19.25 (0.88), 31:25	College students	Correlation analyses	Chapman Psychosis-Proneness Scales (Revised Social Anhedonia, Physical Ahedonia, Magical Ideation and Perceptual Aberration scales)	Siemens Verio, 3T, Visual theory of mind and empathy task		Theory of mind condition Positive correlation SocAn Score-right cuneus, bilateral middle temporal gyrus, medial frontal gyrus and right temporo-parietal junction (all *p* values < 0.05) Positive correlation PAS Score-left middle temporal gyrus (*p* < 0.05) Empathy condition Positive correlation SocAn Score-right cuneus, middle temporal gyrus (all *p* values < 0.05)	
Yin et al., 2015 [79]	15 High SocAn, mean age = 32.00 (12.75), 7:8 15 Low SocAn, mean age = 30.27 (10.47), 5:10	Community sample	Between-group comparisons	Revised Social Anhedonia Scale High SocAn Mean Total Score = 24.60 (5.63) (1.96 SD above the population mean) Low SocAn Mean Total Score = 2.67 (2.53) (equal to or less than 1 SD above the population mean)	3T Siemens Tim Trio Facial emotion processing	Condition Positive vs. Neutral Emotions High SocAn ↓ FC left VLPFC and left inferior parietal cortex, left precentral gyrus/motor cortex, bilateral inferior temporal sulcus, right superior temporal sulcus vs. Low SocAn (all p_FWE_ < 0.05)		
Chan et al., 2016 [80]	8 High Anhedonia, mean age = 18.88 (1.81) 8:0 20 CG, mean age = 19.2 (1.77) 11:9	Community sample	Between-group comparisons	Chapman PAS and SocAnh scales High Anhedonia SocAn Mean Total Score = 10.13 (4.29), PAS Mean Total Score = 31 (6.02) CG SocAn Mean Total Score = 7.75 (4.08) and PAS Mean Total Score = 16.65 (3.66)	Magnetom Verio Siemens 3T monetary incentive delay task (MID) and affective delay task (AID)	AID task Positive vs. Neutral affective Cues High Anhedonia ↓ left thalamus, left thalamus/pulvinar, right insula vs. CG (*p*_FWE_ < 0.005)		
Yan et al., 2016 [81]	18 High POS SCT, mean age = 19.28 (1.18), 9:9 15 High NEG SCT, mean age = 19.33 (1.23), 8:7 22 CG, mean age = 19.78 (0.80), 11:11	College students	Between-group comparisons and correlation analyses	SPQ, PAS, SocAn High SCT SPQ total score > 10th percentile of sample CG SPQ total score < 50th percentile of sample	Siemens Trio, 3T, Monetary Incentive Delay task	Gain vs. non-gain consummation High NEG SCT ↓ right postcentral gyrus, left parahippocampus gyrus/amygdala, the left culmen vs. CG (all *p* values < 0.05, AlphaSim corrected) High NEG SCT ↓ left putamen vs. CG (*p* = 0.0109 AlphaSim corrected) High NEG SCT ↓ right postcentral gyrus, left culmen, left precuneus, bilateral precentral gyrus, left parahippocampal gyrus/amygdala vs. High POS SCT (all *p* values < 0.05, AlphaSim corrected) Gain vs. non-gain anticipation High POS SCT ↑ right VLPFC vs. CG (*p* < 0.05, AlphaSim corrected) High NEG SCT ↓ left ventral striatum, left middle temporal gyrus, cerebellar tonsil bilaterally vs. CG (*p* < 0.05, AlphaSim corrected) High NEG SCT ↓ right ventral striatum-ACC, medial PFC, left superior temporal gyrus, right lingual gyrus, right fusiform gyrus, left middle temporal gyrus vs. High POS SCT (*p* < 0.05, AlphaSim corrected)	Gain anticipation Positive correlation SocAn-right anterior insula in SCT participants (*p* < 0.001 multiple comparison corrected)	
Modinos et al., 2017 [82]	22 High UEx SCT mean age = 27.36 (7.61), 11:11 21 Low UEx SCT mean age = 27.00 (5.64), 12:9	Participants recruited through online advertisement	Between-group comparisons	O-LIFE short version-Unusual Perceptual Experiences Subscale High UEx score > 7 Mean UEx Score = 11.59 (4.93) Low UEx score <2 Mean UEx Score = 0.86 (1.01)	General Electric Discovery MR750, 3T, Emotion-processing task	Condition Emotional vs. Neutral Pictures High UEx ↑ caudate (*p*_FWE_ = 0.023) and ACC vs. Low UEx (*p*_FWE_ = 0.051)		
Günther et al., 2017 [83]	18 High SocAn, mean age = 22.50 (2.73), 0:18 19 low SocAn, mean age = 22.42 (2.46), 0:19	Participants recruited through online advertisement and public notices	Between-group comparisons	Chapman Social Anhedonia Scale High SocAn Mean Total score = 10.89 (1.91) (above the 84th percentile) Low SocAn Mean Total score = 0.74 (0.45) (below the 22nd Percentile)	3T Magnetom Trio, Siemens, Masked Face Processing Task	Condition masked sad vs. neutral faces High SocAn ↑ bilateral thalamus, left red Nucleus vs. Low SocAn (both p_FWE_ < 0.005)		
Papanastasiou et al., 2018 [84]	149 High CAPE, mean age = 19.02 (0.76), 50:99 149 low CAPE, mean age = 18.98 (0.74), 84:65	Healthy adolescents from the IMAGEN database	Between-group comparisons	CAPE-42 questionnaire Upper-lower deciles. High CAPE (upper decile) Mean Total Score = 111.64 (21.26) Low CAPE (lower decile) Mean Total Score = 9.54 (4.75)	3T Siemens, Philips, General Electrics, Bruker Adapted monetary incentive delay task (reward, anticipation outcome)	Anticipation High CAPE ↓ right Caudate Head vs. Low CAPE (*p* = 0.01)		
Wang, Li et al., 2018 [85]	34 High Neg SCT, mean age = 19.21 (0.95), 17:17 30 Low Neg SCT, mean age = 19.23 (0.86), 13:17	College students	Between-group comparisons	Chapman Psychosis-Proneness Scales (Revised Social Anhedonia, Physical Anhedonia scales) High Neg SCT Score) > 23 (sum of scores on physical and social anhedonia) Low Neg SCT Score < 23	Siemens Verio, 3T, Facial emotional valence discrimination task	Neutral Faces Condition High Neg SCT ↓ medial PFC, bilateral amygdala vs. Low Neg SCT (both p_FWE_ < 0.05) Fearful Faces Condition High Neg SCT ↓ left amygdala (*p*_FWE_ < 0.049) and ↓ FC right amygdala-medial frontal gyrus vs. Low Neg SCT (*p*_FWE_ < 0.05) Happy Faces Condition High Neg SCT ↓ FC right amygdala-dorsal anterior cingulate cortex vs. Low Neg SCT (*p*_FWE_ < 0.05)		
Schmidt et al., 2019 [86]	47 participants, mean age = 23.4 (3.6), 18:29	Healthy individuals	Correlation analyses	SPQ	Siemens Magnetom Trio, 3T, Social jumping-to-conclusion task		All last faces vs. all previous faces condition Negative correlation constricted affect SPQ subscale-NAc (*p* = 0.029)	
Olano et al., 2020 [87]	25 participants, mean age = 30.56 (10.25), 9:16	University students and technicians	Correlation analyses	O-LIFE short version Mean Total Score = 10.93(4.69)	Siemens Trio, 3T, Auditory emotional task		Low Intelligibility (most degraded) Condition Positive correlation O-LIFE Total Score-right anterior cingulate cortex, right orbitofrontal cortex and left medial temporal gyrus (all *p* values < 0.05) Positive correlation O-LIFE Unusual experiences subscale-right anterior cingulate cortex and left medial temporal gyrus (all *p* values < 0.05)	
Yan et al., 2020 [88]	74 participants, mean age = 23.50 (3.83), 34:40	Not reported	Correlation analyses	SPQ three-factor model SPQ Mean Total Score = 11.00(9.09)	Siemens Tim TRIO, 3T, Social-cognitive task		Neutral Face Condition Positive correlation SPQ DIS factor-right posterior superior temporal sulcus (p_FWE_ = 0.018) Positive correlation SPQ DIS factor-right-left FC of posterior superior temporal sulcus p_FWE_ = 0.038	

Notes: SCT = Schizotypy; CG = Control Group; SPQ = Schizotypal Personality Questionnaire; FC = Functional Connectivity; PFC = Prefrontal Cortex; DTT = Divergent thinking task; VFT = Verbal fluency Letter and Category task; STA = Oxford Schizotypal Personality Scale; FEP = Fist-Edge-Palm task; PLE = Psychotic-Like. Experiences; DLPFC = dorsolateral prefrontal cortex; VLPFC = Ventral Lateral Prefrontal Cortex; ACC = anterior cingulate cortex; DCP = dorsocaudal putamen; NAc = Nucleus Accumbens; SocAn = Social Anhedonia; PAS = Physical Anhedonia; MagId = Magical Ideation; PerAb = Perceptual aberrations; PDI = Peters Delusion Inventory; PLE = Psychosis-Like Experiences; VRP = Ventrorostral putamen; DMN = Default Mode Network; FEW = family-wise error corrected; FDR = False discovery rated corrected; NEG = negative schizotypy; DIS = Disorganized schizotypy, POS = Positive schizotypy; CP = Cognitive Perceptual SPQ factor; INT = Interpersonal sensitivity SPQ factor; UEx = Unusual perceptual experiences subscale; O-LIFE = Oxford-Liverpool Inventory of Feelings and Experiences; CAPE = Community Assessment of Psychic Experiences questionnaire; NAc = nucleus accumbens; PE = Prediction Error; EPQ = Eysenck Personality Questionnaire; STA = Schizotypal Personality Scale; CAPE = Community Assessment of Psychic Experiences Questionnaire; ACC = anterior cingulate cortex; MID = monetary incentive delay task; AID = affective delay task; UnEx = Unusual Experiences O-LIFE factor; ImpNon = Impulsive nonconformity O-LIFE factor; IntAn = introvertive anhedonia O-LIFE factor.

**Table 2:** The characteristics of the selected studies for SPD patients.

Study (Year)	Participants n, Mean Age (SD), Gender (M:F)	Sample	Diagnostic Criteria for SPD Participants	Design	Scanner, Strength, Task	Findings between Group Comparisons	Finding Correlations
Koenigsberg et al., 2005 [89]	Six SPD participants, mean age = 33.0 (10.7), 5:1 Five CG, mean age = 30.0 (4.9), 3:2	Not reported	DSM-IV criteria for SPD; SADS, SID-p	Between-group comparisons	GE Signa LX 8.2.5, 1.5T, Visuospatial working memory task	Maintenance Period-Memory vs. Control Condition SPD ↓ left ventral prefrontal cortex (*p* = 0.034), left superior frontal gyrus (*p* = 0.042), left intraparietal cortex (*p* = 0.016), left posterior inferior frontal gyrus vs. CG (*p* = 0.048) Retention Period-Memory vs. Control Condition SPD ↓left superior temporal gyrus (*p* = 0.036), left posterior inferior frontal gyrus vs. CG (*p* = 0.048)	
Dickey et al., 2010 [90]	16 SPD, mean age = 39.1 (11.0), 13:3 13 CG, mean age = 35.2 (12.3), 9:4	Community participants	DSM-IV criteria for SPD; SCID	Between-group comparisons and correlation analyses	GE Signa, 3T, Prosody identification task	Whole-brain analysis across conditions SPD-frontal, temporal areas and parahippocampus (all *p* values = 0.001) CG-frontal, temporal, parietal, insular regions (all *p* values = 0.001)	Non-significant findings in SPD group (all *p* values > 0.05)
Hazlett et al., 2012 [91]	28 SPD, mean age = 35.9 (11) 16:12 32 CG, mean age = 32.8 (9.7) 12:20	Not reported	DSM-IV criteria for SPD	Between-group comparisons	Siemens Allegra 3T affective picture processing task	SPD ↑ peak response in amygdala following picture onset vs. CG (*p* < 0.05 corrected) SPD slowest peak latency during picture processing vs. CG (*p* = 0.012) SPD ↑ amygdala to novel pictures vs. CG (*p* = 0.009)	
Vu et al., 2013 [92]	15 SPD, mean age = 38.9 (12.8), 13:2 16 CG, mean age = 32.0 (12.0), 11:5	Participants recruited through advertisements on local public transit, print media and websites	SCID	Between-group comparisons	GE, 3T, 2-back visual working memory task and 0-back continuous performance task	0back vs. rest Condition SPD ↓ left postcentral gyrus vs. CG (*p* < 0.05 corrected) 2back vs. 0back Condition SPD ↓ left posterior cingulate gyrus, left superior temporal gyrus (STG)/insula, left middle frontal gyrus vs. CG (all *p* values < 0.05 corrected)	
Stanfield et al., 2017 [93]	20 SPD, mean age = 37.3 (9.4) 14:6 32 CG, mean age = 36.6 (9.5), 22:10	SPD previously participated in the Edinburgh High Risk Study of schizophrenia and also recruited from clinical services. Controls were recruited from participant and investigator acquaintances and the Scottish Mental Health Network research register	DSM-IV criteria for SPD (SCID-II)	Between-group comparisons	GE Medical Systems Signa Scanner 1.5T Social Judgment task	Non-significant findings (all *p* values > 0.05)	
Resting-state fMRI studies							
Study (Year)	Participant N, mean age (SD), gender (M:F)	Sample	Diagnostic criteria for SPD participants	Design	Scanner, Strength	Findings between group comparisons	Finding correlations/ regressions
Zhang et al., 2014 [94]	18 SPD, mean age = 19.7 (0.9), 18:0 18 CG, mean age = 20.3 (0.9), 18:0	University students	DSM-IV criteria for SPD; SID-*p*	Between-group comparisons	Siemens Verio, 3T	Anterior component of Default Mode Network SPD ↑ FC in bilateral superior temporal gyrus and sub-lobar (bilateral putamen and caudate vs. CG Controls ↑ FC in left superior frontal gyrus, left medial frontal gyrus and cerebellum anterior lobe vs. SPD All *p* values < 0.05 with FDR Correction Posterior component of Default Mode Network SPD ↑ FC in posterior cingulate gyrus vs. CG CG ↑ FC cerebellum posterior lobe, right transverse temporal gyrus, left middle temporal gyrus vs. SPD CG ↓ FC in posterior bilateral cingulate gyrus vs. SPD All *p* values < 0.05 with FDR correction	
Zhu et al., 2017 [95]	19 SPD, mean age = 19.98 (0.82), 17:2 17 CG, mean age = 19.71 (0.71), 16:1	Undergraduate students	DSM-IV criteria for SPD; SCID-II, SPQ three-factor model, Symptom Checklist-90 (SCL-90) Controls had a score at low 10% of SPQ total score	Between-group comparisons and correlation analyses	Siemens Trio, 3T	SPD ↓ FC between the (a) right precuneus and bilateral parahippocampus and right middle temporal gyrus and (b) the right parahippocampus and right superior temporal gyrus vs. CG SPD ↑ FC between right precuneus and right middle frontal gyrus vs. CG all *p* values < 0.05 Alphasim correction	Negative correlation SPQ total score-FC between right precuneus and left parahippocampus in SPD (*p* = 0.006) Positive correlation constricted affect SPQ subscale-FC between right precuneus and middle temporal gyrus in SPD (*p* = 0.003)
Szeszko et al., 2022 [38]	45 SPD, mean age = 45.2 (10.9), 35:10 SPD group was also categorized into two subgroups based on upper lower terciles for Total SPQ score 43 CG, mean age = 43.1 (9.9), 32:11	Controls and SPD were recruited from the community surrounding the university	DSM-IV criteria for SPD; SCID I and SIDP-IV SPQ Mean total SPQ for SPD group = 30.1 (14.9)	Between-group comparisons	Siemens Allegra 3T or Siemens Skyra 3T	SPD with low SPQ scores ↑ FC from mediodorsal nucleus of thalamus to the rostral middle frontal cortex vs. SPD with high SPQ scores (*p* = 0.031)	

Notes: SPD = Schizotypal Personality Disorder; CG = Control Group; DSM = Diagnostic and Statistical Manual of Mental Disorders; SPD = Schizotypal Personality Disorder; SCID = Structured Clinical Interview for DSM; SADS = Schedule for Affective Disorders and Schizophrenia; SID-P = Structured Interview for Personality Disorders.

## Data Availability

No new data were created or analyzed in this study. Data sharing is not applicable to this article.

## Supplementary Materials

The following supporting information can be downloaded at: https://www.mdpi.com/article/10.3390/brainsci13040615/s1, Table S1: Quality assessment of studies with the Newcastle–Ottawa Scale adapted for cross-sectional studies.
