# Supplementary material for: Cognitive Processes and Resting-State Functional Neuroimaging Findings in High Schizotypal Individuals and Schizotypal Personality Disorder Patients: A Systematic Review
Source: Brain Sci. 2023 Apr 4;13(4):615. doi: 10.3390/brainsci13040615 (PMC10137138; doi:10.3390/brainsci13040615)
Supplement: Supplementary file 1 [file brainsci-13-00615-s001.zip › brainsci-2273442-supplementary.pdf]

**Table S1**.Quality assessment of studies with the Newcastle-Ottawa Scale adapted for cross-sectional studies.

| Study | Selection Domain | | | | Comparability Domain | | Outcome Domain | | Total quality score |
|---|---|---|---|---|---|---|---|---|---|
| | Representativeness of the sample | Sample size | Non-respondents | Ascertainment of the exposure | Most important Confounding factor is controlled | Additional factor is controlled | Assessment of the outcome | Statistical test | |
| **Corlett & Fletcher (2012)** | 1 | 1 | 0 | 2 | 1 | 0 | 2 | 1 | 8/10 |
| **Ettinger et al. (2013)** | 1 | 1 | 1 | 2 | 1 | 0 | 2 | 1 | 9/10 |
| **Fink et al. (2014)** | 0 | 1 | 1 | 2 | 1 | 1 | 2 | 1 | 9/10 |
| **Hooker et al. (2014)** | 1 | 1 | 0 | 2 | 1 | 1 | 2 | 1 | 9/10 |
| **Huang et al. (2013)** | 0 | 1 | 0 | 2 | 1 | 1 | 2 | 1 | 8/10 |
| **Modinos et al. (2010)** | 0 | 1 | 0 | 2 | 1 | 1 | 2 | 1 | 8/10 |
| **Modinos et al. (2017)** | 1 | 1 | 1 | 2 | 1 | 1 | 2 | 1 | 10/10 |
| **Mohanty et al. (2005)** | 0 | 1 | 1 | 2 | 1 | 0 | 2 | 1 | 8/10 |
| **Olano et al. (2020)** | 0 | 1 | 0 | 2 | 1 | 0 | 2 | 1 | 7/10 |
| **Park et al. (2015)** | 0 | 1 | 0 | 2 | 1 | 0 | 2 | 1 | 7/10 |
| **Rapp et al. (2010)** | 1 | 0 | 0 | 2 | 1 | 0 | 2 | 1 | 7/10 |
| **Schmidt et al. (2019)** | 0 | 1 | 0 | 2 | 1 | 0 | 2 | 1 | 7/10 |
| **Wang et al. (2014)** | 0 | 1 | 1 | 2 | 1 | 1 | 2 | 1 | 9/10 |
| **Wang et al. (2015)** | 0 | 1 | 0 | 2 | 1 | 1 | 2 | 1 | 8/10 |

| Study | Selection Domain | | | | Comparability Domain | | Outcome Domain | | Total quality score |
|---|---|---|---|---|---|---|---|---|---|
| | Representativeness of the sample | Sample size | Non-respondents | Ascertainment of the exposure | Most important Confounding factor is controlled | Additional factor is controlled | Assessment of the outcome | Statistical test | |
| **Wang et al. (2018)** | 0 | 1 | 1 | 2 | 1 | 1 | 2 | 1 | 9/10 |
| **Yan et al. (2016)** | 0 | 1 | 0 | 2 | 1 | 1 | 2 | 1 | 8/10 |
| **Yan et al. (2020)** | 0 | 1 | 1 | 2 | 1 | 0 | 2 | 1 | 8/10 |
| **Chan et al. (2016)** | 1 | 0 | 0 | 2 | 1 | 1 | 2 | 1 | 8/10 |
| **Van der Meer et al. (2013)** | 0 | 1 | 1 | 2 | 1 | 1 | 2 | 1 | 9/10 |
| **Harvey et al. (2007)** | 0 | 1 | 0 | 2 | 1 | 0 | 2 | 1 | 7/10 |
| **Harvey et al. (2010)** | 1 | 1 | 0 | 2 | 1 | 1 | 2 | 1 | 9/10 |
| **Germine et al. (2011)** | 1 | 1 | 0 | 2 | 1 | 1 | 2 | 1 | 9/10 |
| **Günther et al. (2017)** | 1 | 1 | 1 | 2 | 1 | 1 | 2 | 1 | 10/10 |
| **Yin et al. (2015)** | 1 | 1 | 0 | 2 | 1 | 1 | 2 | 1 | 9/10 |
| **Papanastasiou et al. (2018)** | 0 | 1 | 0 | 2 | 1 | 1 | 2 | 1 | 8/10 |
| **Simon et al. (2015)** | 1 | 1 | 0 | 2 | 1 | 1 | 2 | 1 | 9/10 |
| | | | | | | | | | |
| **Kozhuharova et al. (2021)** | 0 | 1 | 1 | 2 | 1 | 1 | 2 | 1 | 9/10 |
| **Waltmann et al. (2019)** | 1 | 1 | 1 | 2 | 1 | 1 | 2 | 1 | 10/10 |

| Study | Selection Domain | | | | Comparability Domain | | Outcome Domain | | Total quality score |
|---|---|---|---|---|---|---|---|---|---|
| | Representativeness of the sample | Sample size | Non-respondents | Ascertainment of the exposure | Most important Confounding factor is controlled | Additional factor is controlled | Assessment of the outcome | Statistical test | |
| Wang, Yan et al. (2015) | 0 | 1 | 0 | 2 | 1 | 1 | 2 | 1 | 8/10 |
| Wang, Ettinger et al. (2018) | 0 | 1 | 1 | 2 | 1 | 1 | 2 | 1 | 9/10 |
| Wang et al. (2020) | 0 | 1 | 1 | 2 | 1 | 1 | 2 | 1 | 9/10 |
| Wang et al. (2022) | 0 | 1 | 1 | 2 | 1 | 1 | 2 | 1 | 9/10 |
| Zhang et al. (2020) | 0 | 1 | 0 | 2 | 1 | 1 | 2 | 1 | 8/10 |
| Zhou et al. (2021) | 0 | 1 | 1 | 2 | 1 | 1 | 2 | 1 | 9/10 |
| Sabaroedin et al. (2019) | 1 | 1 | 1 | 2 | 1 | 1 | 2 | 1 | 10/10 |
| Wang et al. (2016) | 0 | 1 | 1 | 2 | 1 | 1 | 2 | 1 | 9/10 |
| | | | | | | | | | |
| Folley & Park (2005) | 1 | 1 | 0 | 2 | 1 | 1 | 2 | 1 | 9/10 |
| Hori, Ozeki et al. (2008) | 1 | 1 | 0 | 2 | 0 | 1 | 2 | 1 | 8/10 |
| Hori, Nagamine et al. (2008) | 1 | 1 | 0 | 2 | 0 | 1 | 2 | 1 | 8/10 |
| Kobayashi et al., 2021 | 0 | 1 | 0 | 2 | 1 | 1 | 2 | 1 | 8/10 |
| | | | | | | | | | |

| Study | Selection Domain | | | | Comparability Domain | | Outcome Domain | | Total quality score |
|---|---|---|---|---|---|---|---|---|---|
| | Representativeness of the sample | Sample size | Non-respondents | Ascertainment of the exposure | Most important Confounding factor is controlled | Additional factor is controlled | Assessment of the outcome | Statistical test | |
| **Dickey et al., 2010** | 1 | 1 | 0 | 2 | 1 | 1 | 2 | 1 | 9/10 |
| **Koenigsberg et al., 2005** | 0 | 0 | 1 | 2 | 1 | 1 | 2 | 1 | 8/10 |
| **Stanfield et al., 2017** | 0 | 1 | 0 | 2 | 1 | 1 | 2 | 1 | 8/10 |
| **Vu et al., 2013** | 1 | 1 | 0 | 2 | 1 | 1 | 2 | 1 | 9/10 |
| **Hazlett et al., 2012** | 0 | 1 | 0 | 2 | 1 | 1 | 2 | 1 | 8/10 |
| | | | | | | | | | |
| **Szeszko et al., 2022** | 1 | 1 | 1 | 2 | 1 | 1 | 2 | 1 | 10/10 |
| **Zhang et al., 2014** | 0 | 1 | 0 | 2 | 1 | 0 | 2 | 1 | 7/10 |
| **Zhu et al., 2017** | 0 | 1 | 1 | 2 | 1 | 1 | 2 | 1 | 9/10 |
